# Factors associated with acceptance of preventive treatment among older adults with latent tuberculosis infection in Huzhou, Zhejiang province: a cross-sectional study

**DOI:** 10.3389/fcimb.2026.1967858

**Published:** 2026-10-14

**Authors:** Zhongrong Yang, Zhipeng Zu, Xiuxiu Sun, XinYi Chen, Wei Wang, Pingming Zheng, Dong Wen, Feilin Ren, Zhongming Ding, Ming Gan, Hongyan Wang, Sichao Zhang, Yikun Kong, Guojun Jiang, Jianliang Fan, Jiasheng Qin, Jicun You, Meihua Jin, Bin Chen, Peng Zhang, Jianyong Shen

**Affiliations:** 1 Department of HIV/TB Control and Prevention, Huzhou Center for Disease Control and Prevention, Huzhou, China; 2 Department of Tuberculosis Control and Prevention, Zhejiang Provincial Center for Disease Control and Prevention, Hangzhou, China; 3 Department of Disease Control and Prevention, Huzhou Municipal Health Commission, Huzhou, China; 4 Department of HIV/TB Control and Prevention, Wuxing District Center for Disease Control and Prevention, Huzhou, China; 5 Department of HIV/TB Control and Prevention, Nanxun District Center for Disease Control and Prevention, Huzhou, China; 6 Department of HIV/TB Control and Prevention, Deqing County Center for Disease Control and Prevention, Huzhou, China; 7 Department of HIV/TB Control and Prevention, Changxing County Center for Disease Control and Prevention, Huzhou, China; 8 Department of HIV/TB Control and Prevention, Anji County Center for Disease Control and Prevention, Huzhou, China

**Keywords:** latent tuberculosis infection, LTBI, older adults, TPT, tuberculosis, tuberculosis preventive treatment

## Abstract

**Objective:**

To investigate the acceptance of Tuberculosis preventive treatment (TPT) and the factors associated with treatment acceptance among individuals aged ≥60 years that screened positive for latent tuberculosis infection (LTBI) in Huzhou city, Zhejiang Province of China, and to provide scientific evidence for TPT implementation in this population.

**Methods:**

Older adults aged ≥60 years with LTBI were recruited through community-based health examinations and screening of close contacts of active tuberculosis patients. Univariate and multivariate Logistic regression analyses were performed to identify factors associated with TPT acceptance.

**Results:**

Of the 620 participants enrolled, 430 (69.35%) accepted TPT. Among the participants, 354 (57.09%) were male, 192 (30.96%) were aged over 70 years, 593 (95.65%) were married, 499 (80.48%) resided in rural areas, 577 (93.06%) had an education level of junior high school or below, 135 (21.77%) smoked daily or occasionally, and 152 (24.52%) consumed alcohol occasionally or at least twice weekly. Multivariate Logistic regression analysis revealed that participants with a household size of ≤3 persons (aOR: 1.88; 95% CI: 1.15–3.07), an annual household income of ≤150, 000 CNY (aOR: 2.24; 95% CI: 1.33–3.77), and a history of underlying diseases (aOR: 1.86; 95% CI: 1.16–2.99) were more likely to accept TPT.

**Conclusion:**

The TPT acceptance rate among older adults aged ≥60 years with LTBI in Huzhou was relatively high. Acceptance was independently associated with household structure, economic status, and health status. Smaller household size (≤3 persons), lower annual household income (≤150, 000 CNY), and the presence of underlying diseases were independently associated with TPT acceptance in this population. Targeted intervention strategies should be developed for older adults from larger households, those with better economic conditions, and those without underlying diseases to improve overall TPT coverage.

## 1 Introduction

Tuberculosis (TB) remains a major global public health challenge, and the focus of TB control strategies is gradually shifting from passive treatment to active prevention (Nguyen et al., 2024; Sudersanan et al., 2025). The world health organization (WHO) end TB strategy explicitly identifies tuberculosis preventive treatment (TPT) for high-risk populations with latent tuberculosis infection (LTBI) as a key intervention to reduce TB incidence and interrupt transmission (Coleman et al., 2024). Against the backdrop of global population aging, older adults have become both a priority and a challenge in TB prevention and control. Epidemiological data indicate that the reported incidence of active pulmonary TB among individuals aged ≥65 years in China is substantially higher than the national average, and the prevalence increases with age (Cheng et al., 2020; Jiang et al., 2024; Li et al., 2024). This is primarily attributable to age-related immune senescence, a high burden of comorbidities, delayed healthcare-seeking due to atypical symptoms, and an elevated risk of reactivation of prior infection. Consequently, implementing effective preventive interventions for older adults with LTBI is of critical importance for reducing the overall TB burden in China.

Thus, delivering effective TPT to older adults with LTBI represents the “critical last mile” in achieving the goal of ending TB. Research has demonstrated that short-course, efficacious chemoprophylaxis regimens—such as the 3 months of rifapentine+isoniazid (3HP) regimen—possess favorable safety and adherence profiles (Semitala et al., 2024; Li et al., 2026), providing a feasible technical foundation for scale-up in older populations. However, translating effective treatment protocols from “theoretically viable” to “practically implemented”—particularly among older adults—faces substantial real-world barriers. Prior studies have largely focused on the efficacy, safety, and cost-effectiveness of TPT regimens, or broadly examined adherence-related factors in the general population (Ailinger et al., 2007; Zuniga, 2012). When narrowed to the specific subgroup of adults aged ≥60 years, the initial decision-making process regarding whether to accept TPT remains a relatively neglected “blind spot” in the literature. This decision is the prerequisite for initiating all subsequent treatment procedures, yet it is driven by complex, multi-dimensional factors. For instance, older adults may hold negative attitudes toward TPT due to insufficient risk perception, excessive fear of adverse effects, lack of socioeconomic support, barriers to healthcare access, and the deeply ingrained belief that “absence of symptoms equals absence of disease” (Manoharan et al., 2023).

The existing research gaps are twofold: on the one hand, most quantitative studies have not systematically and thoroughly dissected the specific factors that associate with treatment acceptance among older adults and their relative weights; on the other hand, existing evidence largely derives from single institutions or small samples, lacking community-based investigations at a larger scale, thereby limiting generalizability and practical guidance for public health program design. To address these gaps, the present study systematically investigates the following core research question: What are the independent factors associated with the decision to accept TPT among older adults aged ≥60 years with LTBI? By identifying and quantifying key influencing factors, the findings are intended to provide robust evidence for the design of precision-targeted intervention strategies.

## 2 Materials and methods

### 2.1 Study design and participants

A cross-sectional survey was conducted at the municipal level from January to December 2025, integrating community-based health examinations for older adults and screening programs for close contacts of TB patients. Beyond describing the current status of TPT acceptance in this population, the study employed univariate and multivariate Logistic regression models to quantitatively examine the independent associations between multi-dimensional variables—including demographic characteristics (e.g., age, gender), socioeconomic factors (e.g., income, residence), health status (e.g., history of underlying diseases), and behavioral patterns—and the decision to accept TPT. TPT options included both chemoprophylaxis regimen (Pangprasertkul et al., 2025; Li et al., 2026; Villalva-Serra et al., 2026) and Mycobacterium vaccae immunotherapy (immunotherapy regimen) (Xie et al., 2026). The regimen determination process as physician-recommended with informed patient choice, therefore the regimen of assignment was not randomized. Most participants choose chemoprophylaxis regimen of 3H_2_P_2_ due to its short duration and convenient dosing. Meanwhile, immunotherapy regimen provides an alternative for those unsuitable for or reluctant to undergo chemotherapy.

This study employed a cross-sectional design. Participants were individuals aged ≥60 years identified with LTBI in Huzhou, Zhejiang Province, between January and December 2025, recruited through the following channels: (1) the farmer health examination program, which incorporated LTBI screening for eligible older adults during annual health check-ups; and (2) screening of close contacts of active pulmonary TB patients, targeting older adult close contacts.

### 2.2 Inclusion and exclusion criteria

Inclusion criteria were: age ≥60 years; LTBI confirmed by a positive interferon-gamma release assay (IGRA) or tuberculin skin test; clinically evaluated as meeting the medical indications for TPT as recommended by the Chinese Guidelines for Preventive Treatment of Latent Mycobacterium tuberculosis Infection; clear consciousness with basic communication ability; and provision of informed consent.

Exclusion criteria were: confirmed active pulmonary TB or currently receiving anti-TB treatment; contraindications to TPT (e.g., severe hepatic dysfunction, allergy to treatment medications); and severe mental illness or cognitive impairment precluding survey participation.

### 2.3 Data collection

Data were collected through face-to-face interviews conducted by trained investigators using a structured, standardized questionnaire. The questionnaire covered: (1) sociodemographic characteristics, including age, gender, marital status, residence (urban/rural), education level, household size, and annual household income; (2) behavioral and lifestyle factors, including smoking history (never, occasional, daily) and alcohol consumption (never, occasional, ≥2 times per week); (3) health status, including history of underlying diseases (e.g., hypertension, diabetes); and (4) TPT acceptance, defined as whether the participant agreed to initiate TPT (yes/no) after receiving full information from a physician regarding the purpose, regimen, potential benefits, and risks of TPT. TPT acceptance was defined as whether the participant agreed to initiate TPT (yes/no) after receiving full information from a physician. All participants included in the analysis had been clinically evaluated as eligible for TPT with no contraindications; individuals with contraindications to TPT (e.g., severe hepatic dysfunction, allergy to treatment medications) were excluded at enrollment. The ‘no’ category therefore comprised exclusively participants who were medically eligible but personally declined treatment.

### 2.4 Quality control

All investigators received standardized training and used uniform questionnaires and interview scripts. Completed questionnaires underwent logical checks by designated personnel, with follow-up verification for missing or contradictory information. Data were independently double-entered and subjected to consistency checks.

### 2.5 Ethical approval

The study protocol was approved by the Ethics Committee of Huzhou Center for Disease Control and Prevention (Approval No.: 2025Y003). All participants provided written informed consent.

### 2.6 Statistical analysis

Statistical analyses were performed using R software version 4.5.2. Categorical variables were summarized as frequencies (n) and percentages (%), describing baseline characteristics and TPT acceptance. The chi-square (χ²) test was used to compare the distribution of each variable between the TPT acceptance and non-acceptance groups. Univariate and multivariate Logistic regression analyses were performed to identify factors associated with TPT acceptance. In the dependent variable, yes= accepted TPT, no=did not accept TPT. Variables with P < 0.2 in univariate analysis were entered into a multivariate unconditional Logistic regression model using a forward stepwise (likelihood ratio) procedure to identify independent factors associated with TPT acceptance. Results were expressed as adjusted odds ratios (aOR) with 95% confidence intervals (95% CI). A two-sided P < 0.05 was considered statistically significant.

## 3 Results

### 3.1 Baseline characteristics of participants

A total of 1, 667 individuals with LTBI were identified, of whom 629 were aged ≥60 years; after excluding nine individuals with missing data on key variables, 620 participants were included in the final analysis (see **Figure 1**), of whom 430 (69.35%) accepted TPT. 306 participants received chemotherapy regimens (289 cases received 3H_2_P_2_ regimen and 17 cases received other chemotherapy regimens), accounting for 71.16%; 124 participants received Mycobacterium vaccae immunotherapy, accounting for 28.84%. Among the 620 participants, there were 265 (42.74%) close contacts of active tuberculosis patients, and 355 (57.26%) community-based health examinations. A total of 202 participants with history of underlying diseases, including 93 cases with hypertension, 56 cases with diabetes, 42 cases with hypertension & diabetes, and 11 cases with other history of underlying diseases. Among those who accepted treatment, 250 (58.14%) were male, 315 (73.26%) were aged ≤70 years, 407 (94.65%) were married, 347 (80.70%) resided in rural areas, 334 (77.67%) were farmers or migrant workers, 329 (76.51%) were never smokers or had quit, 313 (72.79%) were never drinkers or had quit, 161 (37.44%) had a history of underlying diseases, and 203 (47.21%) had a body mass index (BMI) ≥24 kg/m². Detailed results are presented in **Table 1**.

**Figure 1:**
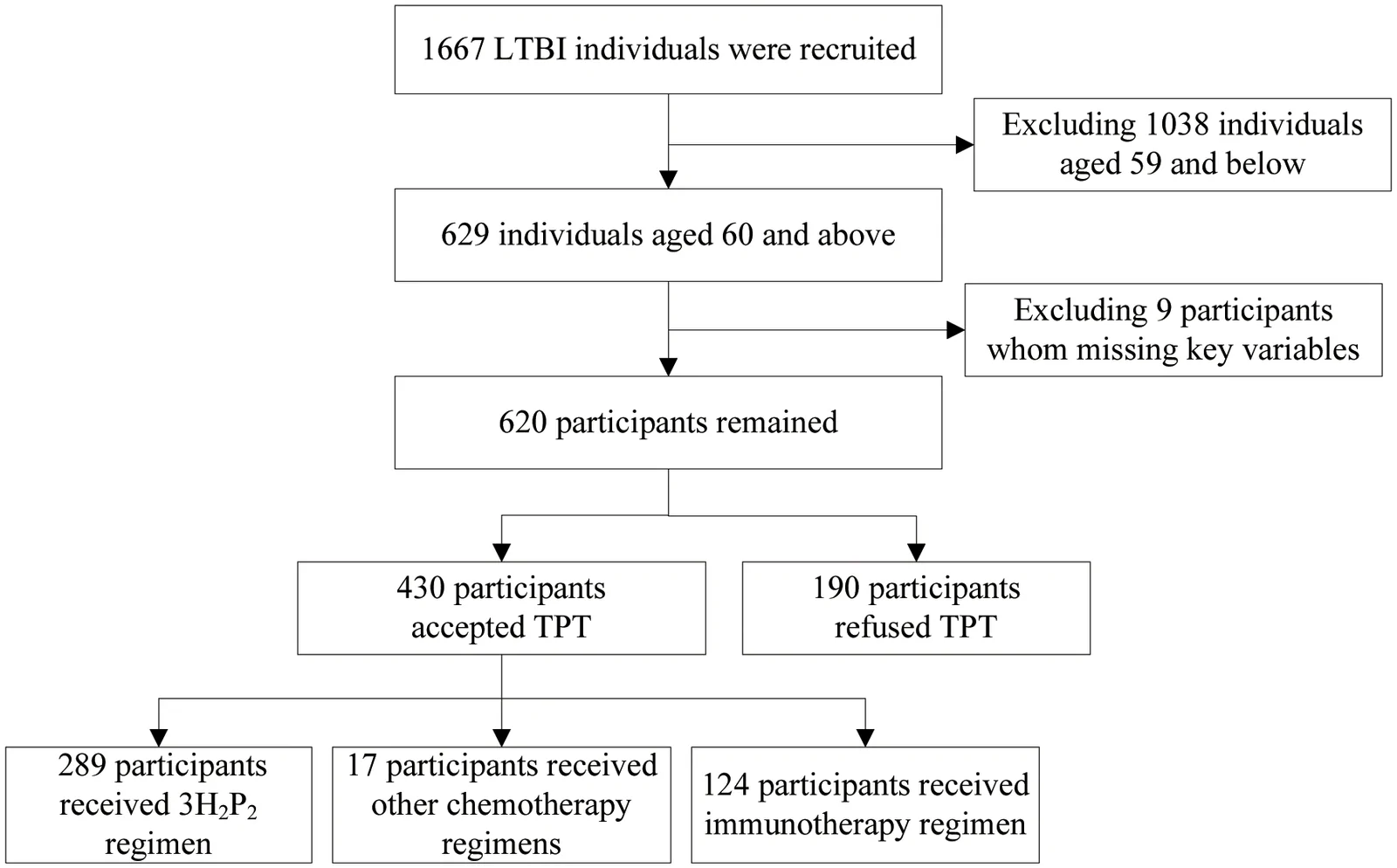
The flow diagram for inclusion of participants.

**Table 1:** Baseline characteristics of participants.

Variable	Category	TPT acceptance		*χ²*	*P*
		No (*n* = 190, *col%*)	Yes (*n* = 430, *col%*)		
Gender					
	Female	86 (45.26)	180 (41.86)	0.492	0.483
	Male	104 (54.74)	250 (58.14)		
Age (years)					
	>70	77 (40.53)	115 (26.74)	11.073	<0.001^**^
	≤70	113 (59.47)	315 (73.26)		
Marital status					
	Unmarried, widowed, or divorced	4 (2.11)	23 (5.35)	2.595	0.107
	Married	186 (97.89)	407 (94.65)		
Residence					
	Urban	38 (20.0)	83 (19.3)	0.008	0.926
	Rural	152 (80.0)	347 (80.7)		
Occupation					
	Other	45 (23.68)	96 (22.33)	0.072	0.789
	Farmer or migrant worker	145 (76.32)	334 (77.67)		
Smoking					
	Never or quit	156 (82.11)	329 (76.51)	2.103	0.147
	Daily or occasional	34 (17.89)	101 (23.49)		
Alcohol consumption					
	Never or quit	155 (81.58)	313 (72.79)	5.035	0.025^*^
	Occasional or ≥2 times/week	35 (18.42)	117 (27.21)		
History of underlying diseases					
	No	149 (78.42)	269 (62.56)	14.382	<0.001^**^
	Yes	41 (21.58)	161 (37.44)		
BMI (kg/m²)					
	<24	124 (65.26)	227 (52.79)	7.846	0.005^**^
	≥24	66 (34.74)	203 (47.21)		

^*^ *P* < 0.05, ^**^P < 0.01.

### 3.2 Factors associated with TPT acceptance

Univariate Logistic regression analysis showed that age ≤70 years (crude OR = 1.87, 95% CI: 1.30–2.67), household size ≤3 persons (crude OR = 1.79, 95% CI: 1.26–2.54), annual household income ≤150, 000 CNY (crude OR = 1.48, 95% CI: 1.05–2.09), alcohol consumption (crude OR = 1.66, 95% CI: 1.09–2.56), history of underlying diseases (crude OR = 2.18, 95% CI: 1.47–3.26), and BMI ≥24 kg/m² (crude OR = 1.68, 95% CI: 1.18–2.40) were positively associated with TPT acceptance (P < 0.05). Conversely, being a close contact of a TB patient (crude OR = 0.20, 95% CI: 0.14–0.28) and having abnormal chest imaging findings (crude OR = 0.62, 95% CI: 0.39–0.97) were negatively associated with TPT acceptance (P < 0.05). Gender, marital status, residence, education level, occupation, and smoking status showed no statistically significant associations in the univariate analysis (P > 0.05).

Variables with P < 0.2 in the univariate analysis—including age, route of identification, marital status, education level, household size, annual household income, smoking status, alcohol consumption, history of underlying diseases, history of TB contact, abnormal chest imaging, and BMI category—were entered into the multivariate Logistic regression model. After adjusting for potential confounders, participants from households with ≤3 persons had an 88% increase in the odds of accepting TPT (aOR: 1.88; 95% CI: 1.15–3.07, P = 0.012); those with an annual household income ≤150, 000 CNY had a 124% increase in the odds (aOR: 2.24; 95% CI: 1.33–3.77, P = 0.002); and those with a history of underlying diseases had an 86% increase in the odds (aOR: 1.86; 95% CI: 1.16–2.99, P = 0.010). Other variables that were statistically significant in the univariate analysis—including age, close contact status, alcohol consumption, BMI, and abnormal chest imaging—lost their independent associations after adjustment for confounders (P > 0.05). Detailed results are presented in **Table 2** and **Figure 2**.

**Table 2:** Univariate and multivariate analyses of factors associated with acceptance of TPT.

Variable	Category	TPT acceptance		Univariate analysis		Multivariate analysis	
		No (*n* = 190, *col%*)	Yes (*n* = 430, *col%*)	*crude OR (95%CI)*	*P*	*Adjusted OR (95%CI)*	*P*
Gender							
	Female	86(45.26)	180(41.86)	*Ref.*			
	Male	104(54.74)	250(58.14)	1.15 (0.81–1.62)	0.430	——	
Age (years)							
	>70	77(40.53)	115(26.74)	*Ref.*		*Ref.*	
	≤70	113(59.47)	315(73.26)	1.87 (1.30–2.67)	<0.001^**^	1.50 (0.96–2.35)	0.076
Population category							
	Community-based health examinations	58(30.53)	297(69.07)	*Ref.*		*Ref.*	
	Close contact	132(69.47)	133(30.93)	0.20 (0.14–0.28)	<0.001^**^	0.28 (0.08–1.04)	0.057
Marital status							
	Unmarried, widowed, or divorced	4(2.11)	23(5.35)	*Ref.*		*Ref.*	
	Married	186(97.89)	407(94.65)	0.38 (0.13–1.12)	0.078	0.35 (0.10–1.19)	0.092
Residence							
	Urban	38(20)	83(19.3)	*Ref.*			
	Rural	152(80)	347(80.7)	1.05 (0.68–1.59)	0.840	——	
Education level							
	Junior high school or below	181(95.26)	396(92.09)	*Ref.*		*Ref.*	
	Senior high school or above	9(4.74)	34(7.91)	1.73 (0.85–3.89)	0.156	2.21 (0.91–5.35)	0.08
Occupation							
	Other	45(23.68)	96(22.33)	*Ref.*			
	Farmer or migrant worker	145(76.32)	334(77.67)	1.07 (0.72–1.61)	0.710	——	
Household size (persons)							
	≥4	119(62.63)	208(48.37)	*Ref.*		*Ref.*	
	less than or equal to3	71(37.37)	222(51.63)	1.79 (1.26–2.54)	0.001^**^	1.88 (1.15–3.07)	0.012^*^
Annual household income (CNY)							
	>150000	94(49.47)	171(39.77)	*Ref.*		*Ref.*	
	Less than or equal to150000	96(50.53)	259(60.23)	1.48 (1.05–2.09)	0.025^*^	2.24 (1.33–3.77)	0.002^**^
Smoking							
	Never or quit	156(82.11)	329(76.51)	*Ref.*		*Ref.*	
	Daily or occasional	34(17.89)	101(23.49)	1.41 (0.92–2.19)	0.121	1.52 (0.84–2.72)	0.165
Alcohol consumption							
	Never or quit	155(81.58)	313(72.79)	*Ref.*		*Ref.*	
	Occasional or ≥2 times/week	35(18.42)	117(27.21)	1.66 (1.09–2.56)	0.019^*^	1.54 (0.86–2.74)	0.143
Core TB knowledge (≥4 correct)							
	No	34(17.89)	83(19.3)	*Ref.*			
	Yes	156(82.11)	347(80.7)	0.91 (0.58–1.41)	0.680	——	
History of underlying diseases							
	No	149(78.42)	269(62.56)	*Ref.*		*Ref.*	
	Yes	41(21.58)	161(37.44)	2.18 (1.47–3.26)	<0.001^**^	1.86 (1.16–2.99)	0.01^*^
History of TB contact							
	Yes	132(69.47)	136(31.63)	*Ref.*		*Ref.*	
	No	58(30.53)	294(68.37)	4.92 (3.42–7.16)	<0.001^**^	2.16 (0.58–8.06)	0.253
Abnormal chest imaging							
	No	151(79.47)	371(86.28)	*Ref.*		*Ref.*	
	Yes	39(20.53)	59(13.72)	0.62 (0.39–0.97)	0.033^*^	0.80 (0.47–1.35)	0.401
BMI (Kg/m^2^)							
	<24	124(65.26)	227(52.79)	*Ref.*		*Ref.*	
	≥24	66(34.74)	203(47.21)	1.68 (1.18–2.40)	0.004^**^	1.29 (0.85–1.94)	0.232

^#^ CNY, Chinese Yuan; ^*^P < 0.05, ^**^P < 0.01.

**Figure 2:**
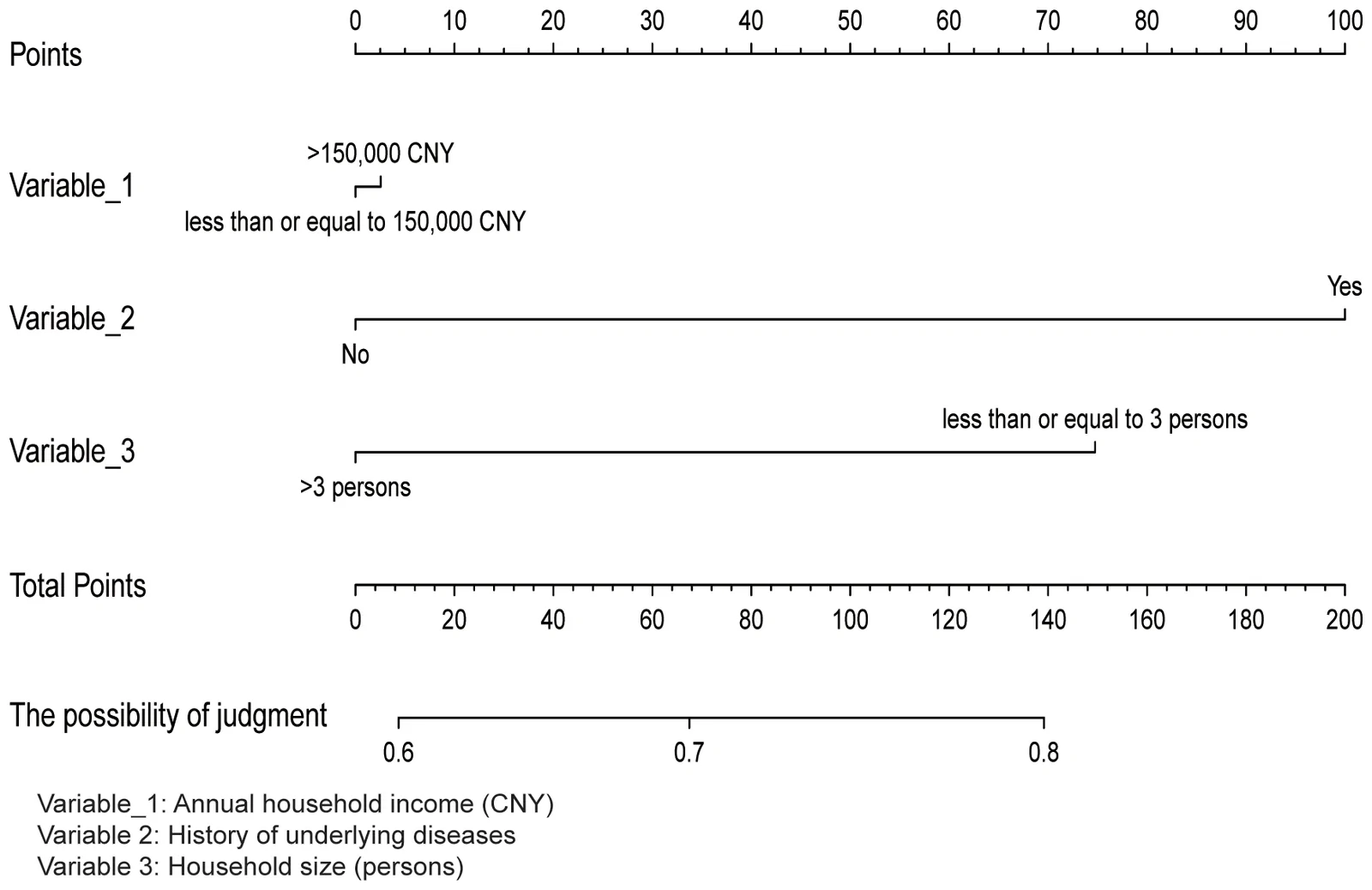
A nomogram of factors associated with acceptance of preventive treatment among older adults with latent tuberculosis infection for the possibility of judgment.

## 4 Discussion

This cross-sectional survey of 620 older adults aged ≥60 years with LTBI in a municipality-level setting systematically examined the current status of TPT acceptance and its associated factors. The overall treatment acceptance rate was 69.35%, indicating a reasonable implementation foundation for preventive intervention. Nevertheless, approximately 30% of the eligible population did not initiate treatment, suggesting that intervention strategies require further optimization. Multivariate analysis identified household structure, socioeconomic status, and health status as the independent core determinants of treatment decisions. The results will assist public health decision-makers and clinicians in shifting from a “one-size-fits-all” promotion model toward more tailored, stratified health education and mobilization strategies, thereby tangibly improving TPT acceptance among high-risk older populations and generating actionable, context-specific scientific evidence for TB prevention and control in older adults, both in China and globally.

Family support and socioeconomic factors play pivotal roles in health-related decision-making among older adults (Yuan et al., 2022; Robles et al., 2023). The present study found that older adults from smaller households (≤3 persons) and those with lower annual household incomes (≤150, 000 CNY) were significantly more likely to accept TPT. The association between lower household income and TPT acceptance warrants careful interpretation. In our multivariate analysis, participants with an annual household income ≤150, 000 CNY were more likely to accept TPT (aOR: 2.24; 95% CI: 1.33–3.77), a finding that appears to contradict the conventional assumption that lower socioeconomic status is generally associated with unfavorable health behaviors. However, we propose three plausible, mutually non-exclusive pathways that may explain this observation in the context of Huzhou. First, the financial barriers that typically link poverty to poor treatment uptake were largely absent in this setting: under the national guidelines for preventive treatment of LTBI and Huzhou’s TB control program, TPT in this study—including medications, baseline examinations, and follow-up monitoring—was delivered free of charge in most or all through the local CDC system at primary healthcare facilities. Second, the opportunity costs of accepting TPT likely differed by income. Our sample was predominantly rural older adults (80.48%), most of whom had exited the labor market, whereas higher-income participants were more likely to remain economically active; for these individuals, the repeated follow-up visits and laboratory monitoring required by TPT may have imposed substantial time and opportunity costs, thereby discouraging acceptance. Third, lower-income participants may have perceived greater vulnerability to disease—consistent with a higher burden of comorbidities—and therefore regarded the freely offered preventive intervention as a worthwhile “health investment” (Yuan et al., 2023; D'Silva et al., 2025). These interpretations should, however, be viewed in light of the cross-sectional design of this study, which precludes determination of temporal ordering. Reverse causation cannot be excluded: it is equally plausible that healthier, economically active individuals declined TPT because of its opportunity costs, whereas those with lower income—often retired and with more flexible schedules—found attendance at follow-up less burdensome. Future longitudinal studies incorporating data on time availability, occupational status, and out-of-pocket costs are needed to disentangle these competing explanations. This finding appears to contradict the conventional assumption that greater resources invariably lead to better health behaviors, but it may instead reflect the unique decision-making logic of older populations. In addition, smaller household size may facilitate more direct communication and concentrated care giving responsibilities, with family members’ attention to and encouragement of the older adult’s health being more effective, thereby promoting TPT acceptance (Alinaitwe et al., 2025). These findings suggest that in health promotion efforts, financial subsidies alone may not be the sole lever; activating intra-family support and risk perception may represent more effective entry points of intervention.

This study confirmed that older adults with a history of underlying diseases were significantly more likely to accept TPT (aOR = 1.86, 95%CI: 1.16–2.99). This finding strongly supports the applicability of the “perceived susceptibility” construct of the health belief model in older populations (Gebremariam et al., 2021). Personal health status is a key intrinsic driver of preventive behavior among older adults. Those with comorbidities (e.g., diabetes, chronic respiratory diseases) may more acutely perceive their health vulnerability and hold stronger perceptions of TB as an infectious threat, thereby showing greater propensity to adopt preventive measures. This finding carries important practical implications: clinicians and public health practitioners may regard older adults with comorbidities as a “priority activation group” for TPT, and enhance their treatment adherence by reinforcing risk communication regarding the interplay between TB and coexisting conditions.

Several variables that were significant in the univariate analysis lost significance in the multivariate model, highlighting the complex interplay among influencing factors. Age ≤70 years, close contact status, alcohol consumption, and higher BMI were associated with TPT acceptance in the univariate analysis, but their independent effects were attenuated after controlling for household, economic, and health-related factors. This suggests that the influence of these variables on treatment decisions may be mediated through, or contingent upon, deeper socioeconomic and health-perception factors. For instance, relatively younger older adults (≤70 years) may be more socially active, have better information access, or bear greater household economic responsibilities—characteristics whose independent age effects are masked when household and economic factors are concurrently present. Close contact status should not be construed as a “barrier”, the crude inverse association may reflects contextual and household differences of the contact subgroup. This carries a practical implication, that is close contacts warrant intensified post-screening mobilization and follow-up education to close the gap in mobilization intensity relative to community-based screening settings. This underscores the need for a systemic perspective when designing intervention strategies, cautioning against simplistic attribution to any single demographic characteristic.

Several limitations of this study should be acknowledged. First, given the cross-sectional design, the temporal ordering of the observed associations cannot be established, and no causal inference should be drawn; future longitudinal studies are warranted to comprehensively evaluate the full spectrum of intervention outcomes. Second, data were primarily sourced from health examination programs and close-contact screening projects, potentially excluding community-dwelling older adults who did not participate in these programs, which may introduce selection bias. Third, the study relied on structured questionnaires without qualitative data on the deeper motivations, concerns, and family interaction processes underlying older adults’ treatment decisions, limiting mechanistic understanding. Because contraindicated individuals were excluded, the factors identified in this study pertain to the willingness to accept TPT among medically eligible individuals rather than to medical suitability—an important distinction that makes the findings directly applicable to the design of TPT mobilization strategies targeting eligible populations. Therefore, the results of this study should not be generalized to older adults with LTBI who have contraindications to TPT. In addition, this study examined only the initial decision to accept TPT; subsequent treatment completion, adverse effects, and long-term preventive outcomes were not tracked. This was a single-center cross-sectional study conducted in Huzhou, an economically developed city in eastern China with a well-established primary TB control network and relatively high insurance coverage and reimbursement levels; our findings may not be directly generalizable to less developed regions. In areas with lower healthcare accessibility, poorer affordability of TPT medications, and less extensive health education, the associations of economic factors (e.g., household income) with TPT acceptance may be stronger; in regions with more nuclear family structures and greater population mobility, the effects of household size may also differ from those observed here. The direction and magnitude of the identified associations therefore require validation in other settings.

In summary, this study delineates a complex landscape of factors influencing TPT acceptance among older adults with LTBI. Future interventions should transcend simple information dissemination models and instead adopt integrated strategies: for individuals from larger households, incorporate family mobilization components into health education; for those with better economic conditions and without underlying diseases, strengthen personal risk perception; and leverage the intrinsic motivation of those with comorbidities, transforming them into active advocates for community-based prevention. Through such precision-targeted, multi-level interventions, TPT coverage among this critical older population may be further improved, thereby solidifying the foundation for achieving the goal of ending TB.

## 5 Conclusion

Through multivariate analysis, this study identified the core structure of factors associated with older adults’ decisions to accept TPT. Smaller household size (≤3 persons), lower annual household income (≤150, 000 CNY), and the presence of underlying diseases emerged as independent factors of treatment acceptance in this population. These findings suggest that older adults in smaller households—where family support may be more concentrated and health vigilance possibly higher—and those with comorbidities—who may be more sensitive to personal health risks and more inclined toward prevention—exhibit greater treatment acceptance. Conversely, the apparent barriers observed at the univariate level, such as older age and close contact status, may be confounded by household and socioeconomic factors. These findings provide an evidence base for developing precision-targeted intervention strategies. Future efforts should prioritize older adults from larger households, those with better economic conditions, and those without underlying diseases, by strengthening health education, optimizing service accessibility, and enhancing social support to improve their willingness to accept treatment.

## Data Availability

The original contributions presented in the study are included in the article/supplementary material. Further inquiries can be directed to the corresponding authors.

## References

[B1] Ailinger R. L. Black P. Nguyen N. Lasus H. (2007). Predictors of adherence to latent tuberculosis infection therapy in Latino immigrants. J. Community Health Nurs. 24, 191–198. doi: 10.1080/07370010701429637 17650988 PMC2603275

[B2] Alinaitwe B. Shariff N. J. Madhavi Boddupalli B. (2025). Treatment adherence and its association with family support among pulmonary tuberculosis patients in Jinja, Eastern Uganda. Sci. Rep. 15, 11150. doi: 10.1038/s41598-025-96260-8 40169867 PMC11961641

[B3] Cheng J. Sun Y. N. Zhang C. Y. Yu Y. L. Tang L. H. Peng H. . (2020). Incidence and risk factors of tuberculosis among the elderly population in China: a prospective cohort study. Infect. Dis. Poverty 9, 13. doi: 10.1186/s40249-019-0614-9 32005290 PMC6995187

[B4] Coleman M. Lowbridge C. du Cros P. Marais B. J. (2024). Community-wide active case finding for tuberculosis: Time to use the evidence we have. Trop. Med. Infect. Dis. 9, 214. doi: 10.3390/tropicalmed9090214 39330903 PMC11436250

[B5] D'Silva O. A. Lancione S. Ananthakrishnan O. Addae A. Shrestha S. Alsdurf H. . (2025). The catastrophic cost of TB care: Understanding costs incurred by individuals undergoing TB care in low-, middle-, and high-income settings - A systematic review. PloS Glob. Public Health 5, e0004283. 10.1371/journal.pgph.0004283 PMC12005564 40173113

[B6] Gebremariam R. B. Wolde M. Beyene A. (2021). Determinants of adherence to anti-TB treatment and associated factors among adult TB patients in Gondar city administration, Northwest, Ethiopia: based on health belief model perspective. J. Health Popul. Nutr. 40, 49. doi: 10.1186/s41043-021-00275-6 34838120 PMC8626924

[B7] Jiang H. Chen X. Lv J. Dai B. Liu Q. Ding X. . (2024). Prospective cohort study on tuberculosis incidence and risk factors in the elderly population of eastern China. Heliyon 10, e24507. doi: 10.1016/j.heliyon.2024.e24507 38314308 PMC10837496

[B8] Li J. Wu Q. Chen W. Wang Y. Xie S. Li T. . (2026). Impact of three-month tuberculosis preventive treatment (3HP) on IVF-ET outcomes in infertile women with tuberculosis infection: a retrospective before-after study. BMC Pregnancy Childbirth 26, 164. doi: 10.1186/s12884-026-08725-x 41645093 PMC12906052

[B9] Li Z. Zhang L. Liu Y. (2024). Analysis of the epidemiological trends of Tuberculosis in China from 2000 to 2021 based on the joinpoint regression model. BMC Infect. Dis. 24, 1223. doi: 10.1186/s12879-024-10126-4 39478490 PMC11526656

[B10] Manoharan A. Siti Nur Farhana H. Manimaran K. Khoo E. M. Koh W. M. (2023). Facilitators and barriers for tuberculosis preventive treatment among patients with latent tuberculosis infection: a qualitative study. BMC Infect. Dis. 23, 624. doi: 10.1186/s12879-023-08612-2 37740196 PMC10517541

[B11] Nguyen T. A. Jing Teo A. K. Zhao Y. Quelapio M. Hill J. Morishita F. . (2024). Population-wide active case finding as a strategy to end TB. Lancet Reg. Health West. Pac. 46, 101047. doi: 10.1016/j.lanwpc.2024.101047 38827931 PMC11143452

[B12] Pangprasertkul S. Buawangpong N. Pinyopornpanish K. Jiraporncharoen W. Angkurawaranon C. (2025). Treatment completion and safety profile of once-weekly 3HP regimen for tuberculosis preventive treatment in children and adolescents: a systematic review. BMC Infect. Dis. 25, 436. doi: 10.1186/s12879-025-10832-7 40155893 PMC11954253

[B13] Robles M. C. O'Brien A. Islam N. McBride A. C. Corches C. L. Mansour M. . (2023). Exploring social determinants of health in healthy aging among older adults: A qualitative study. Prog. Community Health Partnersh 17, 71–78. doi: 10.1353/cpr.2023.0011 37462576 PMC12085860

[B14] Semitala F. C. Kadota J. L. Musinguzi A. Welishe F. Nakitende A. Akello L. . (2024). Comparison of 3 optimized delivery strategies for completion of isoniazid-rifapentine (3HP) for tuberculosis prevention among people living with HIV in Uganda: A single-center randomized trial. PloS Med. 21, e1004356. doi: 10.1371/journal.pmed.1004356 38377166 PMC10914279

[B15] Sudersanan H. Ravikumar N. M. Mishra B. (2025). Estimation of second line anti-tubercular drug susceptibility to Mycobacterium tuberculosis in clinical isolates. Iran. J. Microbiol. 17, 936–941. doi: 10.18502/ijm.v17i6.20361 41510050 PMC12777629

[B16] Villalva-Serra K. Barreto-Duarte B. Rodrigues M. M. Queiroz A. T. L. Croda J. Rolla V. C. . (2026). Programmatic impact and cost-effectiveness of scaling up the short-course 3HP regimen for tuberculosis preventive treatment in Brazil. BMJ Glob. Health 11, e021546. doi: 10.1136/bmjgh-2025-021546 42020107 PMC13110594

[B17] Xie C. Yin P. Song L. Xiong Z. Bai L. (2026). Epidemiological investigation of a tuberculosis cluster outbreak at a university - Changsha City, Hunan Province, China, September 2023-November 2025. China CDC Wkly 8, 520–525. 10.46234/ccdcw2026.084 PMC13187657 42170013

[B18] Yuan B. Zhang T. Li J. (2022). Family support and transport cost: understanding health service among older people from the perspective of social-ecological model. Arch. Public Health 80, 173. doi: 10.1186/s13690-022-00923-1 35850919 PMC9295433

[B19] Yuan Y. Jin J. Bi X. Geng H. Li S. Zhou C. (2023). Factors associated with refusal of preventive therapy after initial willingness to accept treatment among college students with latent tuberculosis infection in Shandong, China. BMC Infect. Dis. 23, 38. doi: 10.1186/s12879-023-08005-5 36670356 PMC9857917

[B20] Zuniga J. A. (2012). Medication adherence in Hispanics to latent tuberculosis treatment: a literature review. J. Immigr Minor Health 14, 23–29. 10.1007/s10903-010-9393-x 20872072

